# Synthesis and characterization of polylactide/rice husk hydrochar composite

**DOI:** 10.1038/s41598-019-41960-1

**Published:** 2019-04-01

**Authors:** Sabzoi Nizamuddin, Ankit Jadhav, Sundus Saeed Qureshi, Humair Ahmed Baloch, M. T. H. Siddiqui, N. M. Mubarak, Gregory Griffin, Srinivasan Madapusi, Akshat Tanksale, Mohd Imran Ahamed

**Affiliations:** 1 School of Engineering, RMIT University, Melbourne, 3000 Australia; 2 Department of Mechanical Engineering, Ahmedabad Institute of Technology, Ahmedabad, Gujrat 380060 India; 3 Institute of Environmental Engineering and Management, Mehran University of Engineering and Technology, Jamshoro, 76090 Sindh Pakistan; 4 Department of Chemical Engineering, Faculty of Engineering and Science, Curtin University, 98009 Sarawak, Malaysia; 5 Department of Chemical Engineering, Monash University, Clayton, VIC 3800 Australia; 6 Department of Chemistry, Faculty of Science, Aligarh Muslim University, Aligarh, 202002 India

## Abstract

Polymer composites are fabricated by incorporating fillers into a polymer matrix. The intent for addition of fillers is to improve the physical, mechanical, chemical and rheological properties of the composite. This study reports on a unique polymer composite using hydrochar, synthesised by microwave-assisted hydrothermal carbonization of rice husk, as filler in polylactide matrix. The polylactide/hydrochar composites were fabricated by incorporating hydrochar in polylactide at 5%, 10%, 15% and 20 wt% by melt processing in a Haake rheomix at 170 °C. Both the neat polylactide and polylactide/hydrochar composite were characterized for mechanical, structural, thermal and rheological properties. The tensile modulus of polylactide/hydrochar composites was improved from 2.63 GPa (neat polylactide) to 3.16 GPa, 3.33 GPa, 3.54 GPa, and 4.24 GPa after blending with hydrochar at 5%, 10%, 15%, and 20%, respectively. Further, the incorporation of hydrochar had little effect on storage modulus (G′) and loss modulus (G″). The findings of this study reported that addition of hydrochar improves some characteristics of polylactide composites suggesting the potential of hydrochar as filler for polymer/hydrochar composites.

## Introduction

Concerns about a future scarcity of petroleum-based resources^1,2^ and environmental degradation caused by use of such sources^3^ have drawn attention towards substitution with bio-based materials in a variety of applications. The concept of blending (either by polymerization or physical^4^) of two materials is in practice since last two decades^5^. Driven by the development of polymer science and technologies^6^, renewable resources-based polymer composite materials are under consideration due to characteristics such as improved sustainability, carbon sequestration and energy efficiency of production, and light-weight^7^. The reinforcement of biomass-based materials using biochar or hydrochar as a filler for synthesis of biomass-based polymer composites is of great interest in polymer research recently. Research has been conducted for incorporation of biochar in different polymer matrices to improve their thermal, mechanical and electrical properties. Das *et al*.^8^ fabricated wood biochar polymer composites and investigated the effect of biochar loadings on the mechanical properties of the composites. It was concluded that the addition of 6 wt.% biochar did not improve the characteristics of the composite, although it was not deleterious either when compared to the properties of the control sample. Composites made by the addition of biochar at 12 wt.% and 18 wt.% were the most ductile and the most thermally stable respectively. The composite made with 24% biochar filler improved the mechanical properties i.e. moduli, flexural and tensile strengths. Although the scientific literature is replete with studies on utilizing biochar as filler in polymer matrices for synthesis of biochar/polymer composite, to date there is no any study reporting hydrochar as filler for polymer composites.

Hydrochar is a carbon-based material synthesized by hydrothermal carbonization of biomass or its derivatives at mild conditions^9^. Hydrothermal carbonization is a promising carbonization method as it is inexpensive, less energy intensive and, as it uses water as a reaction medium, wet biomass can be utilized in the process^10^. Further, chemical usage, pollution and the cost of the hydrothermal carbonization method is comparatively lower than other traditional techniques^9,11^. Hydrochar produced by HTC contains 80–90% of the energy content of the original feed and 55–90% mass of the original mass of biomass^12^. Research on hydrocar synthesis from hydrothermal carbonization is at an early stage and most studies have focused mainly on the effect of parameters used for the carbonization on the yield and the characteristics of hydrochar produced^13^. The applications of hydrochar include; as a carbon material’ an amendment of soil; a solid fuel, which is comparable to brown coal for production of energy; a substitute for activated carbon or carbon black; a carbon catalyst used for production of fine chemicals; a material to increase the efficiency of fuel cells, and; as an absorbent to increase fertility and productivity of soil^14–22^. More studies are focusing on its application as an adsorbent, but these incur several shortcomings. For example, low porosity and surface area of hydrochar causes low adsorption capacity due to inadequate binding sites for attaching to adsorbate^23,24^. The hydrochar also contains polar superficial functional groups which tend to lower the adsorption of non-polar organic matter. Furthermore, hydrochar has lower stability which suggests that hydrochar is not a long-term and recyclable adsorbent^13^. Therefore, this study proposes a novel application of hydrochar as filler in polymer composites.

Rice husk is considered as an agricultural residue coming from rice industry. The lignocellulosic composition of rice husk is dependent of various factors including agronomic handling, weather conditions and soil type^25^. The reported lignocellulosic composition of rice husk is cellulose 35%, hemicellulose 30%, lignin 18%, silica 13% and miscellaneous components upto 4%^26^. Around 140 million tonnes of rice husk are generated annually throughout the world, which is not properly utilized. This huge amount of rice husk is either discarded or burnt in open fields, which are environmentally hazardous strategies^27^. Production of hydrochar (having a number of applications) from rice husk is a way to suggest its effective utilization.

The main objective of this study was to utilize hydrochar produced from microwave hydrothermal carbonization of rice husk as filler for the development of polylactide/hydrochar biocomposites. Polylactide/hydrochar composites were prepared using extrusion techniques at various loadings (5%, 10%, 15% and 20%) of hydrochar in polylactide. The neat polylactide and polylactide/hydrochar biocomposite were analysed for mechanical, thermal, structural and rheological properties. The thermal properties were measured using thermogravimetric analysis (TGA) and modulated differential scanning calorimetry (MDSC), the mechanical properties were analysed through an Instron 4467 universal testing machine, the structural characterization were carried out using scanning electron microscopy (SEM), Fourier transform infra-red (FTIR) spectroscopy, and x-ray diffraction (XRD) analysis and an advanced rheometric expansion system (ARES) was used for rheological testing of polylactide/hydrochar composite.

## Results and Discussion

### SEM analysis of polylactide/hydrochar bio-composites

**Figure 1:**
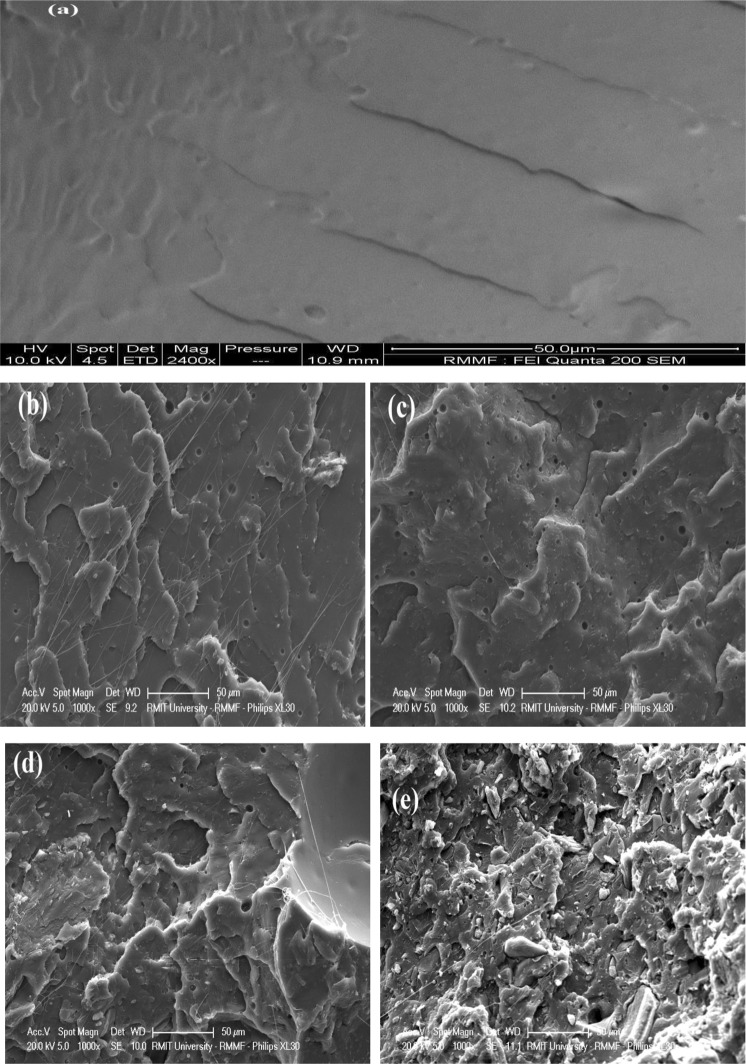
SEM images of (**a**) neat polylactide (**b**) Polylactide/hydrochar-5%, (**c**) Polylactide/hydrochar-10%, (**d**) Polylactide/hydrochar-15%, and (**e**) Polylactide/hydrochar-20%.

The SEM images of the tensile fractured surfaces of neat polylactide and polylactide/hydrochar composites are presented in Fig. 1(a–e). It can be observed that the neat polylactide had a smooth surface whereas the polylactide/hydrochar composites showed fractured surfaces with irregular voids and cracks. The porous structure of char particles allows some of the polymer matrix to penetrate into the char pores^28^. The fracture of composites resulted due to load transfer from polylactide to char particles^29^. From images of polylactide/hydrochar composites, it is observed that the polymer flows through pores of the hydrochar (Fig. 1(d,e)) causing a network of mechanical bonding which results in improved mechanical properties of the composite^30,31^. A high degree of mechanical interlocking/bonding suggests that the char particles are evenly distributed in the polymer matrix^32^. Das *et al*.^33^ also witnessed the mechanical interlocking between polypropylene and biochar. Further, the char particles were well-embedded in the polylactide causing an enhancement in the modulus of the composites. This is attributed to the small particle size of the char and the superior compatibility properties of the char with the polylactide.

### XRD analysis of polylactide/hydrochar bio-composites

**Figure 2:**
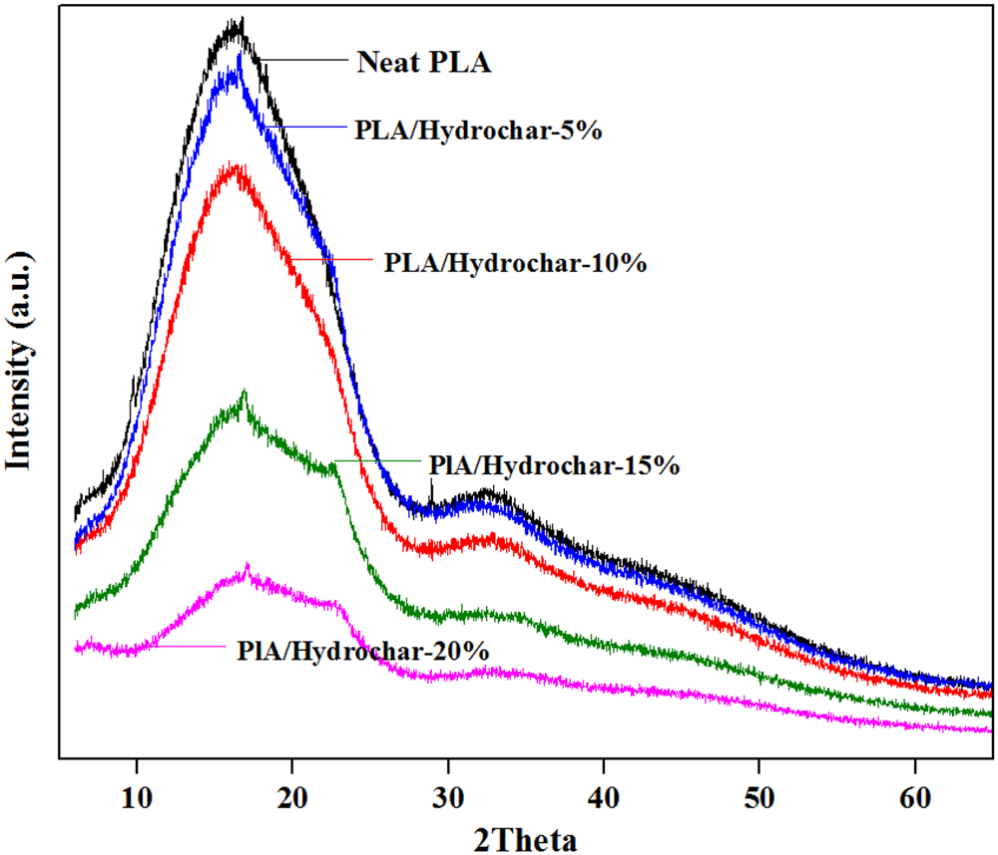
XRD patterns for neat polylactide and polylactide/hydrochar composites.

XRD analysis aids in the analysis of crystalline regions present in the sample. The XRD analysis patterns for neat polylactide and polylactide/hydrochar composites are presented in Fig. 2. Polylactide is a bio-based polymer; therefore it contains random arrangement of both the crystalline and amorphous phases^34,35^. Several peaks are observed at 2θ for neat polylactide which are attributed to the crystalline phase regions existing in polylactide^36,37^. The crystalline structure of the polylactide/hydrochar composites is not altered even at higher hydrochar loadings suggesting that the hydrochar is not modifying the crystalline phases of polylactide and the crystallinity of the composite is mostly conferred by the parent polymer^38^. Further, the peak intensity was decreased by addition of hydrochar and the reduction in peak intensity was greater at higher loadings of hydrochar. This is attributed to the increase of proportion of hydrochar (an amorphous component) and decline of polylactide (a crystalline component) resulting in an increase in the degree of amorphousity in the polylactic polymer^29,38^. The crystallization component of polymer decreases when increasing the amount of char particle in the composite^39^.

### FTIR analysis of polylactide/hydrochar bio-composites

**Figure 3:**
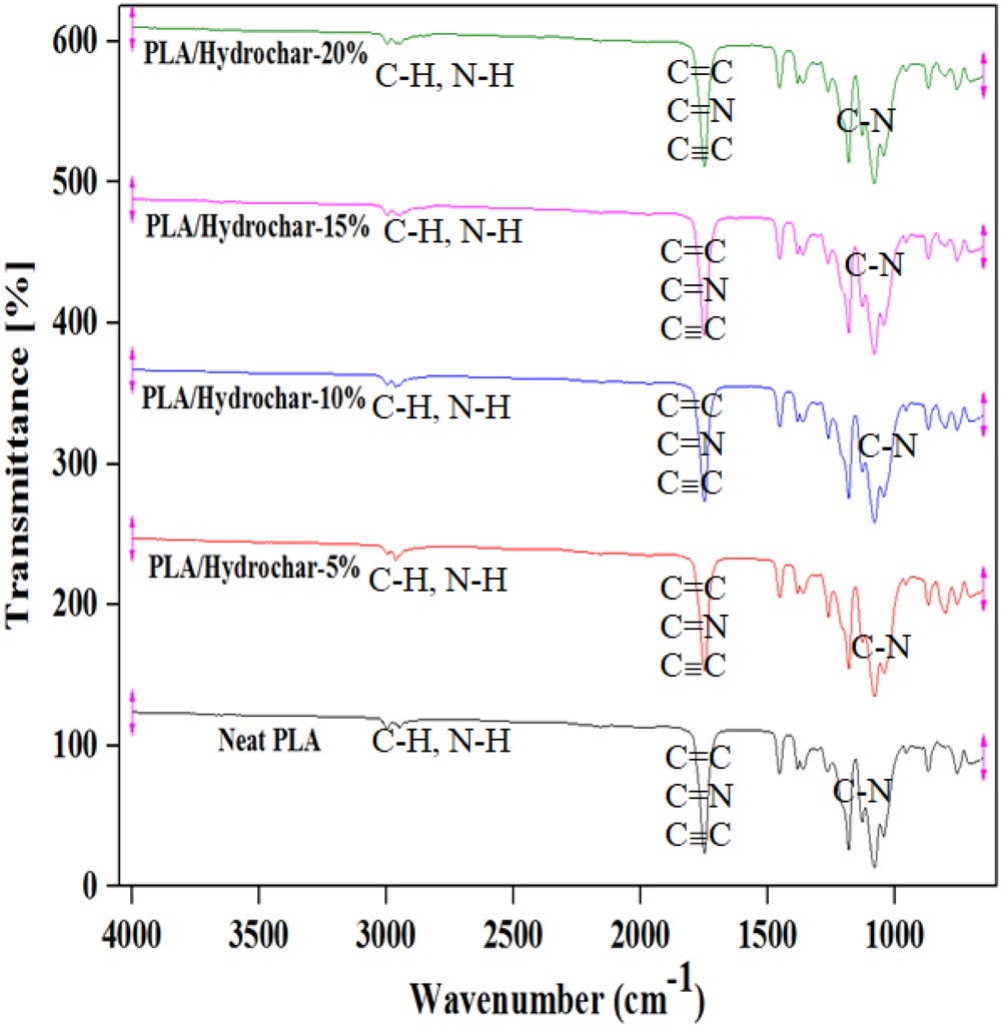
FTIR spectra showing the functional groups present on the surface of neat polylactide and polylactide/hydrochar composites at different loadings.

The FTIR analysis of polylactide/hydrochar composites was carried out to investigate the chemical linkage of hydrochar to polylactide matrix through any copolymerization reaction. The FTIR spectra spectra in the range of 4000–550 cm^−1^ for both the neat polylactide and polylactide/hydrochar composites are shown in Fig. 3. It is evident from Fig. 3 that all the samples had similar spectra and there were neither new peaks formed nor peaks removed in the composites suggesting that chemical interaction did not occur^31^ and only physical mixing occurred. Further, it reveals that the hydrochar addition doesn’t have any particular effect on the molecular structure of the polylactide. Similar results for the FTIR spectra for biochar/polypropylene composites are reported in the literature^40^.

The neat polylactide and polyactide/hydrochar biocomposites exhibited a peak at 1750 cm^−1^, which is attributed to ‒C=O of ester groups^41^. This peak corresponds to strong stretching vibration of ester carbonyl groups^42^ and it is assigned to ‒C=O present in the amorphous phase of polylactide. Moreover, it is noted that the peak at 1750 cm^−1^ is broadening slightly at increasing hydrochar loading suggesting that complexation occurs between polylactide matrix and filler^43^. A peak was observed at 1452 cm^−1^ for neat polylactide and polylactide/hydrochar composites which represents a typical CH_3_ symmetrical bend^44^. The neat polylactide had peaks at 1037, 1085, 1128 and 1182 cm^−1^ which are associated to C-C and C-O stretching. All polylactide/hydrochar blends has peaks at similar positions (with 3 cm^−1^). The peaks detected in the range of 1200-950 cm^−1^ are attributed to stretching of C-O-C, C-O, and C-OH functional groups^45^.

### TGA analysis of polylactide/hydrochar bio-composites

**Figure 4:**
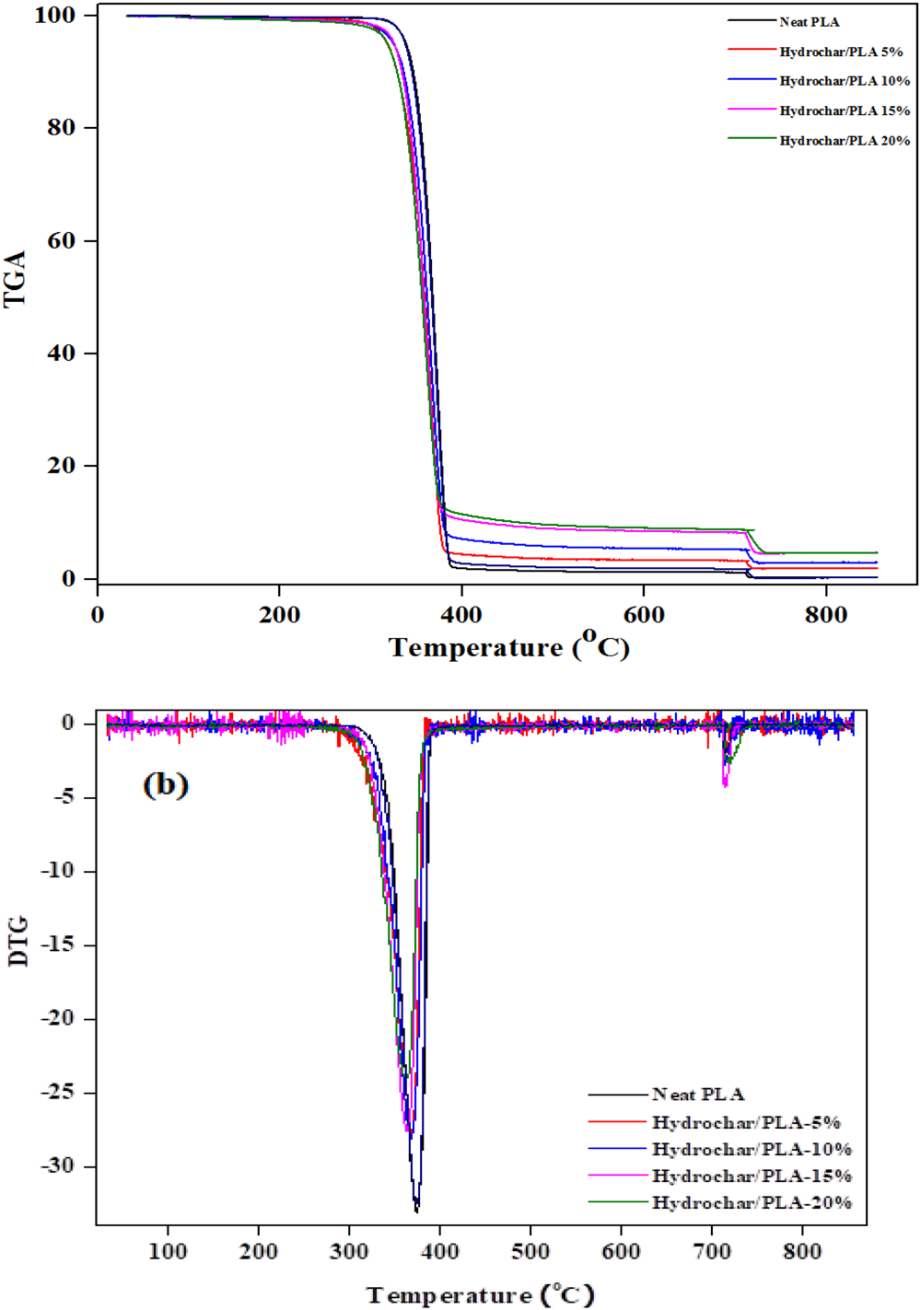
(**a**) TGA analysis and (**b**) DTG analysis of neat polylactide and polylactide/hydrochar composites.

Thermal degradation behaviour is considered as an important and easy tool to measure thermal stability of polymers and their composites during thermal processing^46^. Thermogravimetric analysis (TGA) is generally used to determine the thermal stability and degradation temperature of composite materials^47,48^. TGA analysis curves of polylactide/hydrochar composites are represented in Fig. 4. It is evident from Fig. 4 that the neat polylactide has higher degradation temperature than polylactide/hydrochar composites. Further, it is observed that the composites with higher hydrochar loadings have lower decomposition temperature. It was anticipated that the thermal degradation of composites would start at higher temperatures due to the higher thermal stability of hydrochar. However, this early degradation may be attributed to presence of greater amounts of polylactide in composite^8^. The maximum thermal degradation rate of neat polylactide was at about 390 °C, which decreased to lower temperatures at around 350 °C for all the biocomposite samples. Although degradation temperature of PLA/hydrochar composites was slightly lower than that of neat PLA, they will remain stable in the processing and application ranges of i.e. 30–240 °C of neat PLA without risking thermal degradation^49,50^. All the PLA/hydrochar composites possessed thermal degradation temperature in the range of 350–375 °C (Fig. 4b).

It can be seen from Fig. 4 that neat polylactide left the least residue yield whereas the composites yielded higher amounts of residue which may be attributed to the presence of thermally stable hydrochar in the composite. Ikram *et al*.^30^, studied the thermal stability behaviour of neat polypropylene and polypropylene\biochar composites and found similar results - that the neat polypropylene left lower residue yield than polypropylene/biochar composites after completion of thermal treatment. Another study also reported that the weight percentage of residue increased by increasing the char content in the composites, indicating that the chars may exhibit a condensed phase flame retardant mechanism^46^. The residue produced after the temperature cycle is proportional to the amount of char loaded in the composite, thus the addition of biochar in composites is beneficial when the amount of residue is the main concern^31^.

### MDSC analysis of polylactide/hydrochar bio-composites

MDSC analysis determines several characteristics of polymers including cold crystallization temperature (Tc), enthalpy of crystallization (**∆**H_c_), melting temperature (Tm), melting enthalpy (**∆**H_m_), change of heat capacity (∆Cp), and percentage crystallinity (Xc)^51^. The results of MDSC analysis of neat polylactide and polylactide/hydrochar composites are listed in Table 1. It can be seen from Table 1 that the neat polylactide has a crystallization temperature at 93 °C, which slightly increased after addition of hydrochar due to the nucleation effect of hydrochar particles^40^. It is reported that these particles can act as nucleation sites causing crystal growth, which results in enhancement of the crystallization temperature^30,31^. An increment in the number of particles of char supports earlier crystallization of the polymers^31^. Similar observations have been reported where addition of different types of biochars produced from various feedstocks caused a shift in crystallization temperature to higher values than that of the neat polymer matrix^31^. Crystallization enthalpy was decreased with increasing hydrochar content.

**Table 1:** MDSC analysis of neat polylactide and polylactide/hydrochar composites.

Sample	T_c_ (°C)	∆H_c_ (Jg^−1^)	T_m_ (°C)	∆H_m_ (Jg^−1^)	∆ C_p_ J/(g·°C)	X_c_(%)
Polylactide-Neat	93.1	7.0	168.01	41.60	0.3	37.2
Polylactide/hydrochar-5%	93.8	5.6	167.02	15.99	0.2	17.2
Polylactide/hydrochar-10%	94.4	4.9	166.25	12.78	0.1	16.4
Polylactide/hydrochar-15%	94.6	4.2	166.13	10.99	0.1	16.0
Polylactide/hydrochar-20%	94.9	3.8	166.70	10.44	0.1	16.3

The melting temperature for neat polylactide and polylactide/hydrochar composites at different loadings (5%, 10%, 15%, and 20%) was found to be 168.01 °C, 167.02 °C, 166.25 °C, 166.13 °C and 166.70 °C, respectively suggesting that the hydrochar incorporation in polylactide matrix had little effect on melting temperature. The results of the current study are in agreement to a previous study that biochar addition does not have significant effect on melting temperature^52^. Melting enthalpy of neat polylactide was 41.60 J/g, which was significantly reduced to 15.99 J/g, 12.78 J/g, 10.99 J/g, and 10.44 J/g at 5%, 10%, 15% and 20% loadings of hydrochar, respectively. The variation in melting enthalpy at different hydrochar loadings may be linked to a transformation of crystalline properties of the composite^53^.

The total crystallinity was significantly reduced by addition of the hydrochar in the polylactide matrix, further it was noted that at higher hydrochar loading lower total crystallinity was observed. The decreased total crystallinity of polylactide/hydrochar composites compared to neat polylactide is attributed to the agglomerating nature of hydrochar. The agglomerating nature of the char does not allow free movement of polymer chains, deterring polylactide segments to be packed orderly into an organized crystal form^54^.

### Mechanical characteristics of polylactide/hydrochar bio-composites

**Figure 5:**
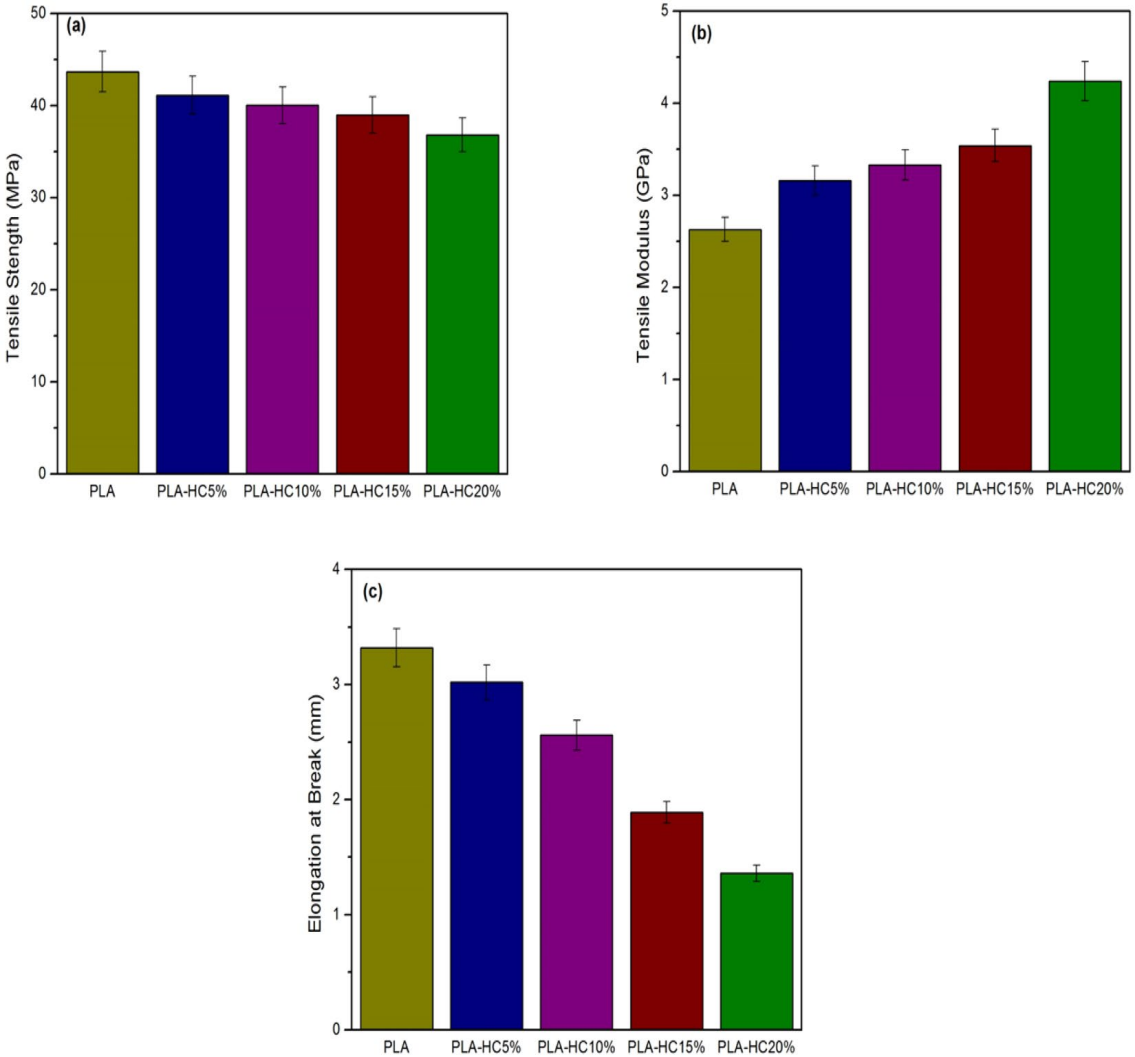
Mechanical properties of neat polylactide and polylactide/hydrochar composites (**a**) tensile strength, (**b**) tensile modulus, and (**c**) elongation at break.

Mechanical performance of the composites is dependent on several factors such as adhesion between filler and polymer matrix, aspect ratio of reinforcing filler particles, crystallinity of the matrix, and volume fraction and orientation of fibers (mostly lignocellulosic and its derivatives)^55^. Figure 5(a–c) shows the mechanical properties of neat polylactide and polylactide/hydrochar composites. It can be observed from Fig. 5(a) that the tensile strength decreases by incorporating hydrochar in the polylactide matrix. Decrement in tensile strength of polylactide/hydrochar composite may be attributed to poor interfacial bonding between polylactide and hydrochar. This also might be due to weak regions of polymer matrix-hydrochar filler, where loops of various chains are in close proximity but are not entangled with each other^56^. Such aggregates of chain ends may cause occurrence of micro-cracks at interfaces lowering the interaction between matrix and filler^57^. Further, a higher rate of decrease in tensile strength is noted at higher loadings of hydrochar. The tensile strength of neat polylactide was found to be 43.69 MPa, which decreased to 41.12 MPa, 40.03 MPa, 38.98 MPa and 36.82 MPa at 5%, 10%, 15% and 20% loading of hydrochar respectively. Lower tensile strength at higher loadings of hydrochar is possibly due higher concentration of hydrochar resulting in the presence of higher amounts of hydrochar interfaces with the polymer matrix^58^. Relatively greater particle distribution of char and low adhesion within polymer matrix resulted in decreased tensile strength at higher loadings of char^59^. The tensile strength can be improved through compatibilization between polymer matrix and char particles^44^. A decline in tensile strength of polypropylene/biochar composite is reported in literature^40^.

The tensile modulus of polylactide/hydrochar composites exhibited an increasing trend with hydrochar loadings as shown in Fig. 5(b). It can be observed that neat polylactide has lower tensile modulus which increases gradually with an increasing content of hydrochar. According to Das *et al*.^33^ a higher modulus of biochar/polymer composite is endorsed by the high surface area of the biochar, which boosted stress transfer between biochar particles and polymer matrix consequently decreasing the deformability of the polymer and improving the modulus. A higher modulus also might be attributed to improved adhesion between the polymer matrix and filler resulting in less and smaller gaps between interfacial surfaces^60^. Nan *et al*.^59^, suggests that an improvement in tensile modulus of a composite is due to higher rigidity of the biochar filler. The gradual increase in tensile modulus of biochar/polypropylene biocomposite with increasing biochar content in the polymer matrix has been reported in literature^40,61^. The elongation at break is illustrated in Fig. 5(c), which reveals that the elongation at break for neat polylactide is higher than polylactide/hydrochar composites. Further, elongation at break decreases with increase in hydrochar loading which is due to aggregation of nonintercalated fillers in the composites enhancing embrittlement^58^. Thellen *et al*.^62^, reported that incorporation of filler into plastics will significantly decrease the elongation.

### Rheological analysis of polylactide/hydrochar biocomposites

**Figure 6:**
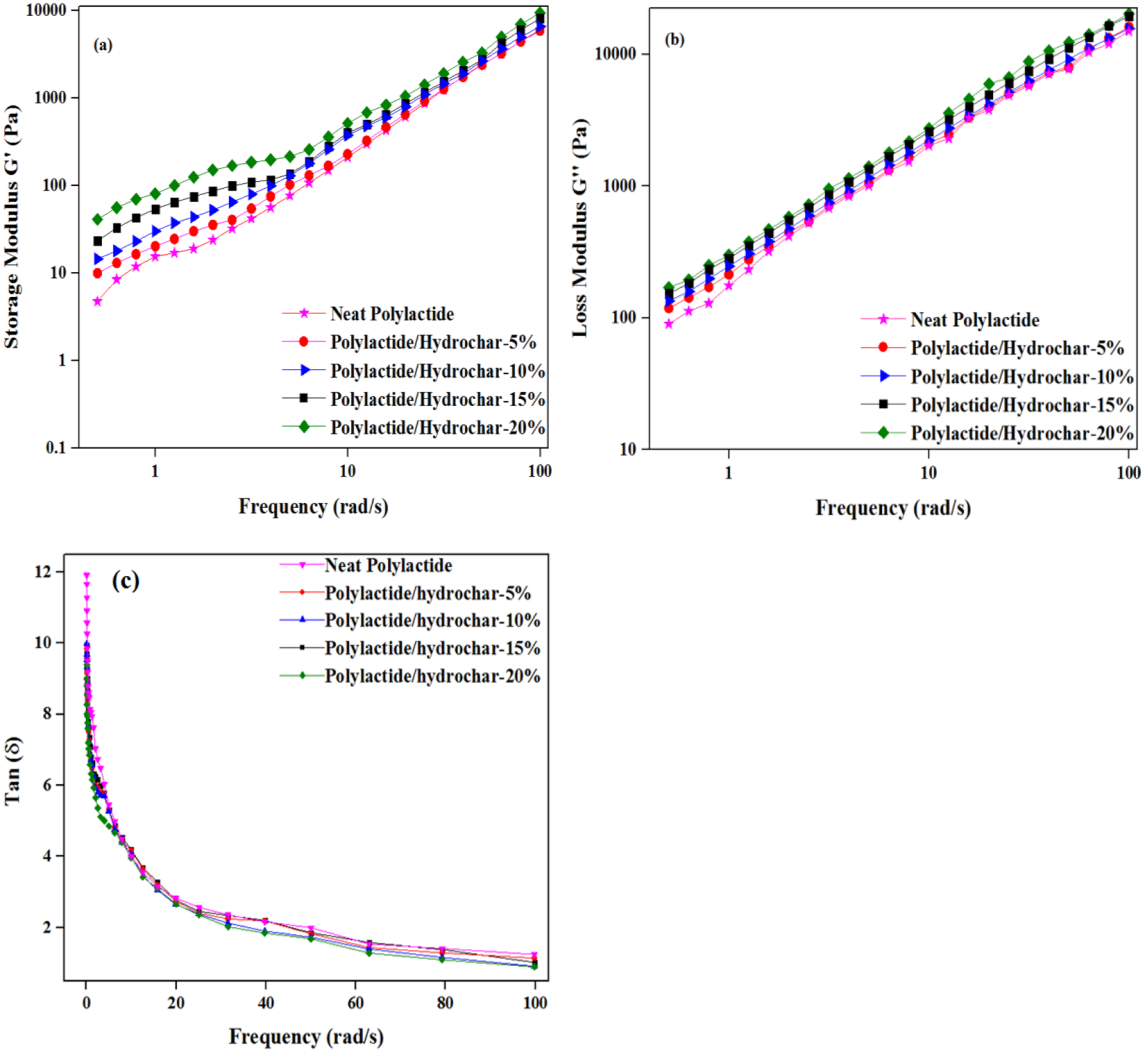
Evolution of rheological characteristics of neat polylactide and polylactide/hydrochar composites at 5%, 10%, 15% and 20% loading of hydrochar: (**a**) storage modulus (G′), (**b**) loss modulus (G″), and (**c**) Tan δ.

Rheological characteristics of the polymers and their composites tend to differentiate the degree of dispersion^51^. The rheological testing was carried out based on dynamic frequency sweep tests at 170 °C and 0.05–100 rad/s. The storage modulus (G′), the loss modulus (G″), and the ratio of loss modulus (G″) to storage modulus (G′) i.e. (tan δ) were obtained from rheological measurements and shown in Fig. 6(a–c). Figure 6(a) represents the relationship between angular frequency (ω) and storage moduli (G′) of neat polylactide and polylactide/hydrochar composites at 5%, 10%, 15% and 20% hydrochar loading. It is observed that the storage modulus (G′) of the polylactide/hydrochar composites is slightly higher than that of the neat polylactide which may be attributed to the formation of a filler-polymer network^40^. Further, it is noted that the storage modulus (G′) increases when increasing the hydrochar content in composites because the hydrochar clusters tend to reduce the mobility of polymer chains and thus increases resistance to flow^63^. The trend for G′ in the low frequency range shows that the storage modulus is higher for the composites than PLA, while in the high frequency range the difference in G′ is not significant. An increment in the storage modulus (G′) of composites is not significant, as expected, due to the impure nature of the hydrochar and the weak interaction between hydrochar and polymer. A weak adhesion or interaction between polymers and fillers have been observed in previous studies including biochar composites and highly engineered carbon composites^40,64^. Similar behaviour for loss modulus (G″) was also observed for all the composites with a higher loss modulus (G″) than that of the neat polymer as shown in Fig. 6(b). Tan δ is generally used to determine the viscoelasticity of the materials, which is more sensitive to relaxation changes than the storage modulus and loss modulus^65^. Figure 6(c) represents the frequency dependence of loss tangent (tan δ) by plotting the data of (tan δ) vs. frequency for neat polylactide and polylactide/hydrochar composites. It is noted that the (tan δ) of neat polylactide was higher at low frequency than that of the polylactide/hydrochar composites, whereas the (tan δ) of both the neat polylactide and polylactide/hydrochar composites was similar at higher frequencies. A decrease in tan δ at higher frequency is the typical characteristic of a viscoelastic liquid^65^. Further, the tan δ is decreasing at higher hydrochar loadings, which is attributed to a polymer-filler network^63^. Similar results for tan δ of biochar/polypropylene composites have been reported in the literature^40^.

### Potential applications of char-added composites

A major challenge for any manufacturing industrial system is to decrease the production cost as well as to achieve sustainability and better properties of the product. The incorporation of renewable materials to produce advanced materials promotes environmental sustainability considering that the renewable material will not generate any further waste^33^. Therefore, utilization of chars as filler for synthesis of polymeric composites is an efficient way of waste utilization together with producing an innovative material^66^. The char, being obtained from waste residues, demonstrates an effective waste management strategy to reduce landfill pressure from dumping of wastes. In addition, the reinforcement of char in polymers improves mechanical, thermal, rheological, electrical and other properties of the composites, consequently allowing different applications for the char/polymer composites. Inclusion of char in a polymer matrix addresses waste utilization, reduces the use of synthetic materials, enhances the properties of the product and potentially reduces cost^61^.

Due to the adsorption characteristics, moisture control, and higher anti-microbial characteristics of the char, the char/polymer composite may have potential application in the food packaging industry^29^. As char can be used to control humidity, regulate moisture and absorb radiation from the environment - char based polymer composite may be used to line wine cellars^66^. The inherent thermal stability of chars supports the application of char based composites as flame retardant materials^44,67^. Therefore, char/polypropylene biocomposites can potentially be used in the interior of automobiles and aeroplanes providing fire-resistant and light-weight material properties^31^. The char/polymer biocomposites possessing acceptable stiffness and fire resistance properties have potential applications in aviation, packaging and playground structures^33^. Future research should focus on investigating various sources of chars and to alter various parameters of char including ash content, particle size, and surface chemistry in order to improve its characteristics and make a more competitive filler^68^. The processing conditions for production of char also can affect the physico-chemical properties of the resulting materials^69^. Therefore, more research is needed to investigate that how these factors affect the properties of composites prepared with char particles.

## Conclusion

This study addressed an issue of waste utilization by addition of hydrochar in polymer matrix for synthesis of polymer/hydrochar composites. The higher porosity of hydrochar supports infiltration of polymers into the pores of hydrochar resulting in some better properties. Thus the hydrochar was utilized in polylactide in this study. The polylactide/hydrochar composites were prepared through melting and mixing of neat polylactide and hydrochar using a Haake rheomix at 5%, 10%, 15% and 20% loading of hydrochar.

The findings of the study suggest that addition of hydrochar in polylactide improved the thermal, mechanical, and rheological properties. When tested by TGA, the residue yield of neat polylactide was higher than that of polylactide/hydrochar composites due to the presence of the thermally stable hydrochar in the composites. The tensile moduli of the polylactide/hydrochar composites were greater than that of the neat polylactide. The neat polyalctide had lower storage modulus and loss modulus as compared to the polylactide/hydrochar composites. Further, the SEM analysis showed that the polymer infiltrates through hydrochar pores causing mechanical bonding between polymer matrix and hydrochar which results in improved mechanical properties of the composite such as higher tensile modulus at higher loadings of hydrochar. The hydrochar/polymer composites may have potential application in food packaging industry, aviation, packaging and playground structures, line wine cellars, and as flame retardant material in the interior of automobiles and aeroplanes providing fire-resistant and light-weight material properties.

## Experimental

### Materials

Polylactic acid (PLA- 4032D Nature-Works,) molecular weight M_w_ - 155,000 g/mol^70^, density- 1.24 g/cm^3^) supplied from Sigma Aldrich was used in this study. The hydrochar used in this study was synthesized through microwave hydrothermal carbonization of rice husk and was characterized using techniques previously developed^71^. Rice husk used for hydrochar synthesis was received from Dowens Rice Hulls Pty. Ltd., Victoria, Australia. The rice husk was washed distilled water to remove dirt and impurities, dried in an oven at 105 °C for 24 h and ground to 1–3 mm before using for the experiments. Both rice husk and synthesized hydrochar were characterized and reported in previous work^71^.

### Preparation of polylactide/hydrochar composites

The polylactide/hydrochar composites films were prepared by incorporating hydrochar in polylactide matrix at four different concentrations as shown in Table 2. The percentage of hydrochar loading in polylactide was based on previous study, which suggested that less than 5% and higher than 15% of char loadings do not show any significant effect on phyico-mechanical properties of biocomposites^31^. The hydrochar and polylactide were physically mixed through hopper of Haake Rheomix, which was used to melt-blend the samples at 170 °C and 40 rpm for 5 min. The samples were coded as polylactide/hydrochar-5 wt%, polylactide/hydrochar-10 wt%, polylactide/hydrochar-15 wt%, and polylactide/hydrochar-20 wt% in which the number denoted the concentration (wt%) of hydrochar in polylactide.

**Table 2:** Sample Compositions.

Sample name	Polylactide (wt.%)	Hydrochar (wt.%)
Neat Polylactide	100	0
Polylactide/hydrochar-5%	95	5
Polylactide/hydrochar-10%	90	10
Polylactide/hydrochar-15%	85	15
Polylactide/hydrochar-20%	80	20

### Characterisation of polylactide/hydrochar biocomposites

The morphology of fractured surfaces of neat PLA and polylactide/hydrochar biocomposite was studied by a Philips XL30 SEM. All the SEM images were acquired at accelerating voltage of 15 kv, at magnification of 2000×, at high vacuum mode. The samples were then sputter coated with layer of gold, about 20 nm in 60 sec to control charging. XRD patterns for polylactide/hydrochar biocomposite were obtained by using a Bruker D4 Endeavor X-ray diffractometer in the angular range of 6–90° (2θ) at 40 kV voltages and 35 mA current. The functional groups on the surface of polylactide/hydrochar composites were determined through FTIR spectroscopic studies using a PerkinElmer FTIR spectrophotometer at 4000 to 450 cm^−1^wavelength, 32 scans per sample at a resolution of 4 cm^−1^. The thermal stability of polylactide/hydrochar biocomposites was analysed using a STA 6000, (Perkin-Elmer). The samples were heated from 30 °C to 600 °C at 10 °C min^−1^ heating rate. The samples were held for a minute at both the initial and final temperatures. The testing was repeated three times and sample weight percentage was plotted as a function of temperature.

A DSC-2920 Modulated DSC (TA Instruments) was used to study the thermal properties of the polylactide/hydrochar biocomposites. Initially, the instrument was standardized with indium at 35 ml/min flow rate of nitrogen. An empty aluminium pan wrapped with lid was used as reference. Approximately 7–10 mg of polylactide/hydrochar biocomposite samples were encapsulated in the aluminium pan. For first heating cycle, the samples were heated in the range of −20 °C to 220 °C at 20 °C/min heating rate and then cooled at 2.00 °C/min to −20.00 °C. This scan removed any earlier thermal history of the polylactide/hydrochar biocomposites. For the second heating cycle, the samples were heated from −20 °C to 220 °C at 2 °C/min heating rate and were modulated +/− 0.50 °C every 40 seconds. The lower heating rate for the second heating cycle was employed for accurate tracing of small transitions. The samples were left for five mins at both the initial and final temperatures in both cycles. The degree of crystallinity (X_c_) of neat polylactide and polylactide/hydrochar biocomposites was calculated through Eq. (1):

1 $$Xc=100\times [\frac{{\rm{\Delta }}{H}_{m}-{\rm{\Delta }}{H}_{c}}{{\rm{\Delta }}{H}_{m}^{c}}]\times \frac{1}{{W}_{PLA}}$$

Where ∆H_m_ denotes the melting enthalpy, ∆H_c_ refers to the cold crystallisation enthalpy, ${{\rm{\Delta }}H}_{{\rm{m}}}^{{\rm{c}}}$ is the enthalpy of pure polylactide (93 Jg^−1^)^72,73^ and W_PLA_ is the weight fraction of the polylactide in polylactide/hydrochar biocomposites.

For mechanical testing, the samples were compression-moulded into dog-bone shaped ASTM D638 mechanical testing specimens at 180 °C compression moulding temperature for 5 min while 80 kN of compression force was applied. Further, the moulding press was cooled down to 50 °C with the help of cooling water. The mechanical testing of the samples was conducted through an Instron 4467 universal testing machine according to ASTM D638M at 1 mm/min speed rate. Rheological studies of the polylactide/hydrochar bio-composites were studied by a strain-controlled ARES (TA instruments) rheometer. The bio-composites were tested through a force transducer with 0.2–200 g-cm torque range and 25 mm diameter parallel-plate fixture. Measurements were carried out at 170 °C temperature. Zero gap calibrations were checked at 170 °C before running each test. Linear viscoelastic regions (LVR) of the composites were examined through strain sweep experiments at 1 rad/s frequency and 0.1–100% strain. Further, dynamic frequency sweep tests were conducted again on fresh samples in LVR at 0.05–100 rad/s frequency range for understanding hydrochar loadings effect on storage (G′), loss modulus (G″) and complex viscosity (η*) of the polylactide/hydrochar biocomposites.
